# Mining of transcriptome identifies CD109 and LRP12 as possible biomarkers and deregulation mechanism of T cell receptor pathway in Acute Myeloid Leukemia

**DOI:** 10.1016/j.heliyon.2022.e11123

**Published:** 2022-10-18

**Authors:** EbyNesar StellaGlory Deepak Shyl, Beutline Malgija, Appadurai Muthamil Iniyan, Ramasamy Mahendran, Samuel Gnana Prakash Vincent

**Affiliations:** a International Centre for Nanobiotechnology (ICN), Centre for Marine Science and Technology (CMST), Manonmaniam Sundaranar University, Kanyakumari District, Rajakkamangalam 629502, Tamil Nadu, India; b Computational Science Laboratory, MCC-MRF Innovation Park, Madras Christian College, Chennai 600 059, Tamil Nadu, India; c York Bioscience Private Limited, Ambattur Industrial Estate, Chennai, Tamil Nadu 600 058, India; d Cancer Therapeutics Lab, Department of Microbial Biotechnology, Bharathiar University, Coimbatore, Tamil Nadu 641046, India

**Keywords:** Acute myeloid leukemia, Network analysis, Differential gene expression, Gene ontology, Secretome analysis, Variant analysis

## Abstract

Acute Myeloid Leukemia (AML) is a heterogeneous disease with highest mortality compared to other types of leukemia. There is a need to find the gene abnormalities and mechanisms behind them due to their heterogenic nature. The present study is aimed to understand genes, pathways and biomarker proteins influenced by transcriptomic deregulation due to AML. Differentially expressed gene (DEG), protein-protein interaction network, gene ontology, KEGG pathway, variant analysis and secretome analyses were performed using different AML RNAseq datasets. A total of 655 DEGs including 291 up-regulated and 364 down-regulated genes, which were satisfied with a fold change of 1.5 were identified. Top hub genes for AML were identified as TP53, PTPRC and AKT1. This integrative bioinformatics approach revealed the deregulation of T Cell Receptor (TCR) pathway and altered immune response related genes. The survival analysis revealed the associated deregulation of multiple TCR pathway related genes. Variant analysis identified the benign and likely benign nature of many important target genes and markers screened, which were found to have an important role in the progression of AML. DEGs and secretome analysis found out a set of seven molecules represents potential biomarkers for AML. *In vitro* analytical validation showed overexpression pattern of CD109 and LRP12 in AML cell line and HL-60 cells than the normal human bone marrow-derived stromal cell line HS-5. Here we report first time for CD109 and LRP12 as a possible biomarkers for the diagnostic significance. Amino acid substitutions detected by variant analysis and deregulation of immune checkpoint molecules revealed their role in reducing immune response and inability to fight cancer cells. In conclusion, this study highlights the possibility of new biomarkers for AML and the mechanism of decrease in immune response due to the downregulation of co-stimulatory immune molecules, which needs further clinical validation investigations.

## Highlights

• Using RNA-seq data of AML patients, two biomarkers including CD109 and LRP12 for the diagnostic significance were identified based on DEGs, GO/KEGG, and PPI network analysis.
• The transcriptome mining unmasked the complexity of gene alterations in AML by identifying immune response related genes deregulation and significance of TCR signalling.
• Several genes were identified as AML hub genes by network analysis, variant analysis identified non-synonymous variants in co-stimulatory checkpoint targets and the co-inhibitory targets.

## 1 Introduction

The cancer therapeutic decision-making is largely dependent on molecular pathway deregulations that guide as predictive biomarkers. The Precision Medicine Initiative by National Cancer Institute (NCI) is formed in 2015 aimed to scale up efforts to identify cancer genomic drivers and apply that information in the development of more effective approaches to cancer treatment. The heterogenic nature of cancer needs attention to understand hinges on the development of valid biomarkers interrogating key aberrant pathways potentially targetable with molecular targeted or immunologic therapies [1]. Acute myeloid leukemia (AML) is a phenotypic and genetically heterogeneous disease, categorized by numerous genetic abnormalities and gene mutations. AML is the most dominant form of leukemia in neonatal and adult ages but signifies a small fraction of cases during infancy and adolescence [2]. AML usually starts in the bone marrow, but most often it quickly moves into the blood, as well. At times it can spread to other parts of the body namely lymph nodes, spleen, liver, central nervous system (brain and spinal cord), and testicles. AML diagnosis is mainly based on bone marrow and peripheral blood analysis. The pathophysiology of AML is not yet understood well at the cellular and molecular level, and recently cytogenetic markers are the most important for risk stratification and treatment of AML patients [3]. Targeted sequencing approach has identified numerous mutations that convey prognostic information, including gene mutations in FLT3, NPM1, KIT, CEBPA, and TET2 [4]. Biomarkers play a progressively vital role in the clinical management of cancer patients. World Health Organization suggests that “A biomarker is any substance, structure or process that can be measured in the body or its products and influence or predict the incidence of outcome or disease” [5].

The genomic data richness and computational tools allow us to find specific mutation, pathway deregulation switch and disease progression. Understanding genetic mutations are the important element of AML. For example, mutation in the gene Nucleophosmin 1 (NPM1), a nucleolar phosphoprotein that performs diverse biological functions including molecular chaperoning, DNA repair, ribosome biogenesis, and genome stability are one of the most frequent molecular abnormalities in AML in patients with a normal karyotype [6, 7]. Roughly 12% of AML patients with mutation in the tumour protein p53 (TP53), which is involved in cell cycle arrest and apoptosis [8, 9]. Specific diagnosis is made by immunophenotyping and cytochemistry searching for myeloperoxidase activity in blasts or by immunophenotyping surface markers like CD123, CD45, CD34, CD38 and others [10]. The advent of new in-depth sequencing technologies necessitates the detection of other molecular markers such as point mutations epigenetic and proteomic profiles, have begun to play an important role. A very recent report on transcriptome mining has predicted a novel AML biomarker COMM domain-containing protein 7 (COMMD7) which is involved in the regulation of NF-kappa B signalling [11, 12].

AML patients treated with rigorous chemotherapy, targeted therapy or bone marrow transplantation improved survival [13]. However, despite the understanding of its pathophysiology, mortality rates remain high. For instance, in 2020 there were an estimated 60,530 new leukemia cases and 23,100 deaths in the unites states [14]. The poor outcomes are due to late detection and lack of achieving complete remission [15]. This difficulty insists the urgent need for diagnostic and prognostic markers identification from RNA-seq data. Rapid improvements in high-throughput technologies and omics have led to the identification of novel genetic abnormalities and diagnostic biomarkers of AML. The purpose of the present study is to compare the genes expression changes in AML against normal samples by using statistical analysis and performing functional, pathway enrichment network analyses and to gain insights on the impact of genetic variations on gene deregulation using variant analysis and protein-protein network analysis.

## 2 Materials and methods

### 2.1 Filtration, alignment, batch correction and differentially expressed gene (DEG)

**Figure 1:**
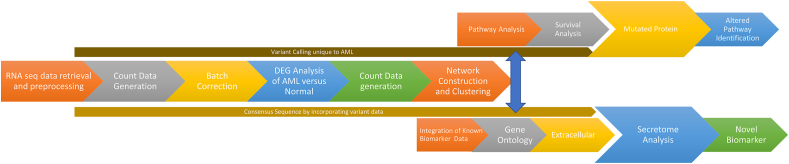
Workflow of the proposed study.

The AML RNA-seq samples used in the study were collected from Sequence Read Archive (SRA) from four different bioprojects with accession numbers PRJEB21548, PRJNA428149, PRJNA576867 and PRJNA390519. Aligned reads and the count data was generated by STAR alignment [16], sorted by SAM TOOLS [17] and the count data were generated by HTSeq count [18]. To overcome the technical and biological differences across different samples batch correction was done by ComBat-seq [19]. In order to find the AML specific gene expression, it is essential to find the genes that are differentially expressed than the normal expression pattern. Differential expression was detected using DESeq2. Rows with only zeroes, those with little to no information regarding the amount of gene expression were removed. The magnitude (log_2_ transformed fold change) and significance (P-value) of differential expression between AML and control were calculated. Genes with a fold change one and false discovery rate (FDR) adjusted P-values <0.05 were counted as differentially expressed. The detailed workflow of the present work is given in Figure 1.

### 2.2 Protein-protein interaction (PPI) network analysis

To examine the interaction and hub genes of DEGs, protein-protein interactomes were constructed using the STRING app of Cytoscape. Network analyzer and CentiScape were used to analyze the topological parameters of the network. Genes with a degree of connectivity >35 were considered hub genes. MCODE module was used to extract the clusters from the network, by setting a node cut-off 0.2 and K-core 2.

### 2.3 Functional annotation of DEGs

The unknown gene symbols were converted using g:convert from g:profiler [20]. G:convert can convert between various gene, protein, microarray probe, RNAseq and numerous other types of namespaces. Functional enrichment was performed using multiple sources, including FunRich, PANTHER, DAVID, VarElect and GeneAnalytics. In GeneAnalytics, the unknown gene symbols were also converted to their respective Ensembl IDs before analysis. Functional (GO, pathway) and expression (Disease, tissue) information of the DEGs were analysed.

### 2.4 Variant calling and analysis

The sorted bam file was further indexed by Tabix before the accomplishment of variant calling by bcftools *mplieup*. For the assessment of variation in the candidate genes and the potent biomarker genes, the consensus sequence was produced by bcftools *consensus*. Based on the consensus sequence and fasta file of GRch38, the gene sequences for the screened DEGs were extracted. The variant from AML and normal samples were merged in bcftools, compared by isec and the variants unique to AML were extracted and annotated in ANNOVAR [21]. This annotates the variants with the RefSeq Genes annotations with reference GRCh38 [22]. RefSeq gives information about the type of mutation and the amino acid change when the variation occurs in the coding region. The variant data of the screened DEGs were analysed for their correlation with clinical impact using Variant Interpretation for Cancer (VIC). VIC uses the pre-annotated files and classifies sequence variants based on numerous criteria, which helps to improve the interpretation on clinical impacts [23]. The functional impact of the amino acid substitution was assessed based on SIFT, Polyphen2, FATHMM and Provean scores of the non-synonymous SNVs.

### 2.5 Survival analysis

The correlation of DEGs related to immune response with overall survival was examined by Kaplan-Meier plotter. The overall survival curves created using KM (Kaplan-Meier) method were produced by reference to the median gene expression levels. We employed the 50% quantiles of gene expression with 95% confidence interval as cut-offs for the KM curves obtained by Leukemia Gene Atlas (LGA) (http://www.leukemia-gene-atlas.org/) which supports analysis of leukemic data.

### 2.6 *In vitro* analytical validation by western blot analysis

AML cell line and HL 60 cells and human bone marrow-derived stromal cell line HS 5 were extracted after 90% confluence without treatment. Total protein lysates were extracted using RIPA buffer supplemented with 1% phenylmethylsulfonyl fluoride (PMSF). Protein concentration was estimated by Bio-rad protein quantification solution. After quantification, 40 μg of protein sample from each group was loaded on SDS-PAGE (10–12%) at a constant 90 V and transferred onto PVDF membrane (Milipore, Bedford, MA. 0.45 μm pore size) using Bio rad transfer apparatus. Blots were then blocked in blocking buffer (5% milk, 20 mM Tris–HCl, pH 7.6, 150 mM NaCl, and 0.1% Tween 20). After TBST washes, blots were incubated in primary antibodies overnight at 4 °C. The immunoblots were rinsed three times in TBST buffer for 10 min each rinse and then incubated in their respective secondary antibodies for 1 h at RT. The membranes were then washed in TBS buffer for 10 min three times. Blots were developed using ECL chemiluminescent reagent and documented. ‘Image J’ software was used to quantify the expression levels of proteins. Nuclear proteins were extracted using NE-PER® Nuclear and Cytoplasmic Extraction Reagents (Thermo Fisher Scientific, Inc. Rockford, USA) according to the manufacturer’s instruction. The following antibodies were used in this study: CD109 (#sc-271085 Santa Cruz), LRP12 (#EPR9056 ab150352 Abcam) and GAPDH (6C5) (sc-32233 Santa Cruz), all secondary antibodies (anti-rabbit and anti-mouse) were purchased from Santa Cruz Biotechnology. All reagents were purchased from Sigma.

## 3 Results

### 3.1 Identification of differentially expressed genes in AML

Publicly available AML specific RNA-seq datasets (Table S1) from SRA database were downloaded and processed through standard pipeline and utilized for the study. The normalized expression of each gene was measured by FPKM (Fragments Per Kilobase of transcript per Million mapped reads). We identified the DEGs among AML comparable to normal samples using DEseq2 and detected 655 genes including 291 up-regulated and 364 down-regulated genes, which were satisfied with a fold change of 1.5 and P-value < 0.05. The top ten up and down regulated genes are depicted in Table 1.

**Table 1:** List of top deregulated genes identified using DEG analysis.

Sl. No	Gene symbol	Description	Biological function	Fold change	P-value
1	EGFL7	EGF-Like Protein 7	Vasculogenesis regulation	2.99	0.00
2	MCL1	Myeloid Cell Leukemia 1	Cell survival	2.99	0.00
3	IGHG1	Immunoglobulin Heavy Constant Gamma 1	Antigen binding	2.39	0.1
4	CD109	CD109 molecule	Negative regulation of TGF-β signaling	2.38	0.00
5	GNA15	G Protein Subunit Alpha 15	Cell signal transduction	2.36	0.00
6	FAM30A	Family With Sequence Similarity 30 Member A	Cell migration	2.21	0.00
7	MEIS1	Meis Homeobox 1	Development, hematopoiesis	2.19	0.00
8	MAP7	Microtubule Associated Protein 7	Cell polarization and differentiation	2.15	0.00
9	SLC17A9	Solute Carrier Family 17 Member 9	Transport of small molecules	2.14	0.01
10	HSPG2	Heparan Sulfate Proteoglycan 2	Endothelial growth and regeneration, vascularization	2.14	0.00
11	CD3E	CD3e molecule	Immune response	-2.74	0.05
12	GNLY	Granulysin	antimicrobial	-2.74	0.01
13	IL32	Interleukin 32	Immune response	-2.69	0.02
14	FGFBP2	Fibroblast Growth Factor Binding Protein 2	Immunity	-2.55	0.01
15	FCMR	Fc Fragment Of IgM Receptor	Immune system processes	-2.47	0.05
16	TCF7	Transcription Factor 7	natural killer cell and innate lymphoid cell development	-2.47	0.00
17	TBX21	T-Box Transcription Factor 21	Developmental process regulation	-2.44	0.00
18	CD2	CD2 molecule	Optimize immune recognition	-2.38	0.00
19	ZAP70	Zeta Chain Of T Cell Receptor Associated Protein Kinase 70	Immune response	-2.32	0.01
20	IL2RB	Interleukin 2 Receptor Subunit Beta	Immune response	-2.27	0.03

### 3.2 Construction and analysis of PPI networks

To further investigate the molecular mechanism behind the pathogenesis of AML and interactive relationships among all DEGs, the DEGs from each analysis were mapped separately to string database and the validated interactions with the confidence score >0.7 were selected to construct the protein-protein interaction (PPI) networks. The 655 DEGs were searched for their biological interaction ability from String database and network was created. Interactions that did not satisfy the above said cut-off were not considered and the constructed network consists of 601 nodes and 1728 edges. The R-squared value of node degree distribution and topological coefficients were found to be in acceptable range for the network. Clustering coefficient resides another important parameter that renders knowledge on the overall organization of the interconnections within a network. This measures the extent to which a node gets clustered and this lies between 0 and 1, which was also observed to be in the preferable range. The genes namely TP53, PTPRC, IL2, AKT1, ITGAM, SYK, RPS27A, LCK, FLT3, UBA52, FYN, CD2, JUN, CD3D, CD28 and ZAP70 were identified as the hub genes and the list of top ten genes with highest degree is displayed in Table 2. Among the hub genes, most of the genes were found to be down-regulated.

**Table 2:** List of high-ranking genes identified using PPI network analysis.

Gene symbol	Name	Family	Type of Expression	Degree	Occupancy in module
TP53	Tumour protein P53	TF	Normal	56	Module VI
PTPRC	Protein tyrosine phosphatase receptor type C	Enzyme	Normal	52	Module III
AKT1	AKT Serine/Threonine Kinase 1	Kinase	Normal	51	Unclustered
ITGAM	Integrin Subunit Alpha M	Integrin	Down	50	Module III
SYK	Spleen Associated Tyrosine Kinase	Kinase	Normal	48	Module I
RPS27A	Ribosomal Protein S27a	Ribosomal protein	Normal	48	Module I
LCK	LCK Proto-Oncogene	Src Family Tyrosine Kinase	Down	48	Module I
UBA52	Ubiquitin-52 Amino Acid Fusion Protein	Ubiquitin, ribosomal	Normal	45	Module I
FYN	FYN Proto-oncogene	Src family tyr kinase	Down	45	ModuleI
JUN	Jun Proto-Oncogene	bZIP	Up	44	Module X1

TF- Transcription factor; N-terminal section of UBA52 belongs to ubiquitin family and C-terminal to ribosomal protein el40 family.

### 3.3 Functional analysis of the network and significant module identification

ClueGO analysis found out functional enrichment and GO terms is shown in figure S1. Annotation of the significant genes in the network showed that most of the genes share their localization in the membrane. Moreover, the network mainly showed the enrichment of immune response-related processes like lymphocyte, leukocyte activation and positive regulation of T-cell receptor (TCR) signaling. The web view of the constructed network and functional annotation can be assessed using the URL: https://sites.google.com/view/acutemyeloidleukemia/home.

**Figure 2:**
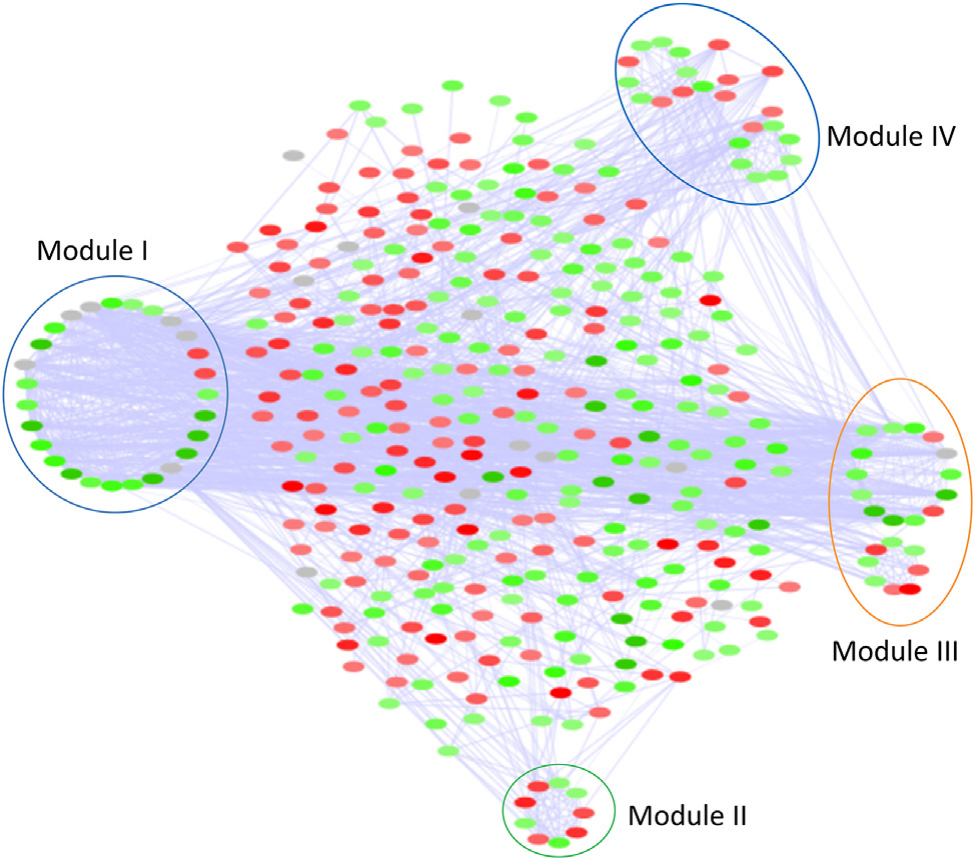
PPI network of the differentially expressed genes in AML. Red and green nodes denote the up and down regulated genes respectively coloured based on their Fold change. Grey denotes the interactive partners from String.

The main PPI network was further analysed for dense regions using MCODE and was ranked according to the density and the number of nodes. Modules with score >5 were assumed to play an important role in the pathologic features of AML. MCODE generated 17 modules from the PPI network; in which 4 clusters were filtered based on the preferred cut-off (Table 3 and Figure 2). Different modules generated by the MCODE clustering algorithm emphasized the deregulation of genes related to immune response and T-cell receptor (TCR) signalling. Module 1 corresponds to genes related to immune response and protein binding. SYK was identified as the seed (highest scoring node) of the cluster. Most of them were found to localize in the plasma membrane. The genes CD2, CD3D, CD3E, CD8A, CD8B and IL6 corresponds to hematopoietic cell lineage occupies the module I. Module II occupies the genes related to platelet degranulation (GAS6, IGFBP7, LAMC1), Endoplasmic reticulum (ER) to Golgi vesicle-mediated transport and cell adhesion (F5, GAS6, SERPINA1). The functions enriched by Module III include TCR signalling, protein ubiquitination and negative regulation of apoptosis. Most of the genes in module III reside to locate in plasma membrane. Module IV is enriched by interferon-gamma (INF-γ) mediated signalling (OAS1, GBP1, IRF2, HLA-F, HLA-DQB1, MT2A) and immune response (OAS1, SAMHD1, LILRB2, HLA-F, HLA-DQB1). Most of the hub genes occupied different modules excluding TP53 and AKT1. Based on their importance in the network 106 genes were screened for further studies.

**Table 3:** MCODE modules of significant AML genes. Red and green denote the up and down-regulated genes respectively. Grey represents their interactive partners from STRING.

Module	Network	Score (S), Nodes (N) & edges (E)	Enriched function
I		S: 12.462 N: 27 E:162	TCR signaling Protein binding hematopoietic cell lineage
II		S: 6.182 N: 19 E:113	Platelet degranulation Cell adhesion ER to Golgi vesicle-mediated transport
III		S: 6.000 N: 12 E:34	TCR signaling Protein ubiquitination Negative regulation of apoptosis
IV		S: 5.556 N: 9 E:24	INF-γ mediated signaling Immune response

### 3.4 Immune deregulation of T cell receptor pathway in AML

Functional annotation and pathway enrichment of the top DEGs were performed following adjustment of the data set to exclude any changes with a p-value of >0.05, gene ontology network pathway analysis of the top up and down-regulated genes were carried out.

#### 3.4.1 Functional enrichment

**Figure 3:**
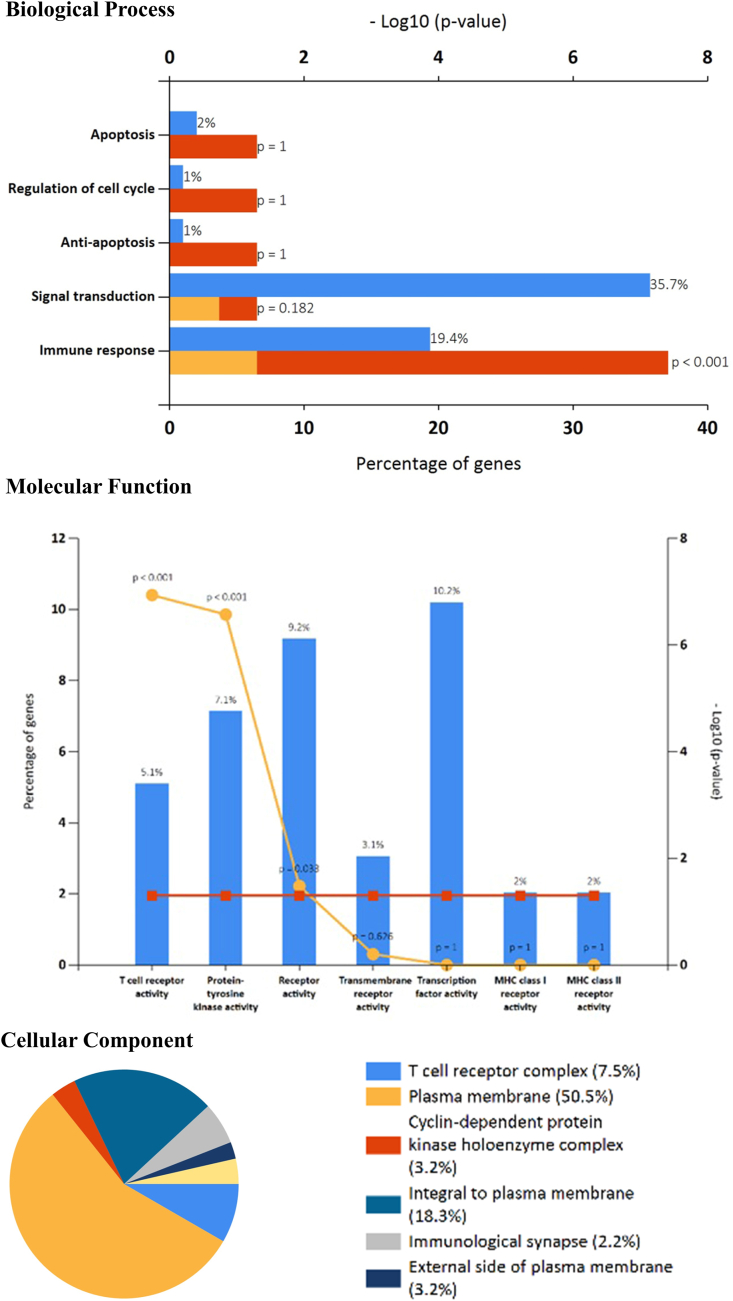
Function enrichment of the significant genes using FunRich. This shows the gene ontology terms Biological process, Molecular function and Cellular component.

Investigation of Gene Ontology (GO) terms biological process and molecular functions related to AML is depicted in Table S2. Go terms found out the matching of 104 genes to a total of 116 GO-Biological process categories. The top hits with high scores include T cell receptor (TCR) signalling pathway (score 41.44) with an alteration of 14 genes out of 179. Similarly, 100 genes were matched with 31 GO-Molecular functions. But only three terms possessed high scores: protein binding (score 48.61) with 93 genes out of total 11,207 genes. In terms of cellular component 100 genes matched to 24 GO terms with the top hits of plasma membrane (score 44.05). Functional enrichment using FunRich also showed the enrichment of similar terms as displayed in Figure 3.

#### 3.4.2 Finding disease causing likelihood genes from the screened genes

Translating the obtained result into significant discoveries by inferring biological and clinical importance is an important step towards analysing high-throughput RNA-seq data. Among the 106 symbols obtained from the previous MCODE analysis, 64 were directly related and 42 symbols were indirectly related to AML. The strength of the connection between the gene highlights FLT3, RUNX1, ETV6, ATM, ZBTB16, PAX5, CDK4, MYB, FANCA, MPO, CBFA2T3, PRF1, CSK, MCL1, CD38, CDKN1A, CD8A, MEIS1, IL3RA, CD5, EZH2, ITGAM, IL3RA, MYB, MCL1, LCK, CD4 and CDK6 as the top genes. VarElect also predicted the average disease-causing likelihood for each gene based on RVIS (Residual variation intolerance score) and GDI (Gene Damage Index). It is calculated based on the principle that a variant in a gene with high mutation intolerance is more likely to be disease-causing. This shows the genes ZBTB16, CDK4, IL1B, JUN, CDK6, ETV6, ARHGEF1, AFDN, MYB, MVP, PLK1, CD28, MEIS1 and MCL1 among the top-scoring genes (score > 200) has high disease-causing likelihood (>60%) ratio. The top genes with indirect relations include BLK, PLCG1, CCR7, CD3E, GNA15, CD247, MT2A, BIN1 and UBA52.

#### 3.4.3 Encoded protein class and KEGG pathway enrichment of DEGs in AML

To further understand the proteins' function of the top DEGs, we examined their protein classes using PANTHER [24]. Encoded proteins of the screened genes were mainly distributed among defence/immunity protein (PC00090), gene-specific transcriptional regulator (PC00264), metabolite interconversion enzyme (PC00262), protein modifying enzyme (PC00095), protein-binding activity modulator (PC00095) and transmembrane signal receptor (PC00197). In addition, the number of proteins associated with immune response, was high, which was consistent with the GO analysis and DEGs identification analysis, suggesting immune response may play an important role in the pathogenesis of AML.

Pathway enrichment of the significant genes using GeneAnalytics uses data mined from PathCards (pathcards.genecards.org), which displays pathways as superpaths merged from twelve sources [25]. This predicted 20 super pathways consistent with them and the top pathways are depicted in Table 4. This shows the significance of genes related to innate immune system, hematopoietic stem cells and lineage-specific markers, TCR signaling, ICos-ICosL pathway in T-helper cell, Class-I MHC mediated antigen processing and presentation, NF-kappaB signaling, Th17 cell differentiation and GPCR pathway. The innate immune system pathway presented the highest match score with 65 genes (score 125.82), followed by haematopoietic stem cells and lineage-specific markers (score 80.95) and T-cell receptor signalling pathway (score 73.02) specific to AML.

**Table 4:** Top enriched super pathways of AML significant genes using GeneAnalytics.

S.No	Pathway	Score	Matched genes (Total genes)	Important gene symbols
1.	Innate Immune System	125.82	65 (2124)	RASAL3, EVL, FGRFLT3, FOS, TNRC6C, IL1B, IL32, RORA, KLRB1, KLRD1, OAS1, RASA3, CAMK4, IGHG1, PSTPIP1CD28, LCK, CSK, PIK3CD, CD8B, IL12RB1, IL18, IL7R, GNLY, ITGB7, CD79B, ITK, CD14, DUSP16, BIN2, STK10, CCL5, HLADQB1, FCGR3A, CARD11, HLAF, YES1, ZBP1, CDKN1A, GBP5, GZMM, ZAP70, CD3E, RASGRP1, CD300E, CD247, CLEC2D, SOCS3, PLCG1, JUN, FCN1, IL3RA, SIGLEC10, CD4, ITGAL, FYN, CD3D, VNN1, LTF, IGHG3.TRAC, MCL1
2.	Hematopoietic stem cells & lineage-specific markers	80.95	20 (116)	FLT3, KLRB1, CD28, MS4A1, SLAMF1, CCR7, CD2, ITGAM, IL2RB, MPO, IL7R, CDS, CD8A, CD69, CD79B, CD14, CD3E, LY9, CD226, IL3RA, CD38, CD4, ITGAL
3.	T Cell receptor signalling pathway	73.02	21 (183)	FOS, IL1B, PSTPIP1, CD28, GRAP2, LCK, PIK3CD, CD8B, FYB1, BATF, CD8A, ITK, CARD11, ZAP70, CD3E, RASGRP1, GAB2, CD247, PLCG1, CDK4, TEC, JUN, SKAP1, CD4, FYN, CD3D, MAP4K1
4.	TCR signalling in Naïve CD4^+^ T cells	72.55	16 (66)	CD28, GRAP2, LCK, CSK, CD8B, PAG1, FYB1, CD8A, ITK, PRF1, CARD11, ZAP70, CD3E, RASGRP1, GAB2, CD247, PLCG1, CD4, FYN, CD3D, MAP4K1
5.	ICos-ICosL pathway in T-Helper cell	72.28	19 (131)	FOS, CD28.GRAP2, LCK, CSK, IL2RB, SYK, IL2, PTPRC, ITK, HLA-DQB1, ZAP70, CD3E, CD247, PLCG1, JUN, CD4, FYN, CD3D
6.	Class I MHC mediated antigen processing and presentation	72.19	34 (823)	KLRB1, BTBD6, CD28, GRAP2, LCK, CSK, CD8B, LILRB2, ZBTB16, UBA52, SYK, BLK, PTPRC, LMO7, RNF213, CD8A, ITK, HLA-DQB1, HLA-F, CDKN1AZAP70, CD3E, ZNRF1, RPS27A, CD247, PLCG1, EGF, SH3RF1, KCTD7, CD4, ITGAL, FYN, CD3D
7.	NF-kappaB signaling	68.72	24 (327)	IL1B, OAS1, CD28, LCKGZMB, CD2, ZBTB16, IL2RB, MPO, MYB, SYKBLK, IL2, PTPRC, RUNX1, CD8A, ITK, ZAP70, TBX21, CDBE, SOCS3, PAXS, IRF2, CD38
8.	Th17 cell differentiation	66.63	19 (162)	FOS, IL1B, CD28, LCK, IL6, IL2RB, IL2, RUNX1, HLADQB1, ZAP70, TBX21, CD3E, CD247, SOCS3, PLCG1, JUN, CD4, CD3D, MCL1
9.	NFAT in Immune response	61.77	18 (162)	FOS, CD28, GRAP2, LCK, UBA52, SYK, PTPRC, ITK, ZAP70, CD3E, CD247, PLCG1, JUN, CD4, FYN, CD3D
10.	GPCR pathway	60.45	29 (712)	FLT3, FOS, ARHGEF1, CD28, LCK, CSK, IL6, IGF2R, ITGAM, SYK, GASO, IL2, ITK, P2RY1, HLA-DQB1, CDKNIA, GNA15, ZAP70, CD3E, E2F1, CD247, PLCG1, EGF, CDK4, JUN, CD4, ITGAL, FYN, CD3D
11.	Hematopoietic cell lineage	58.10	15 (99)	FLT3, IL1B, CD8B, IL6, CD2, ITGAM, IL7R, CD5, CD8A, HLA-DQB1, CD3E, IL3RA, CD38, CD4, CD3D
12.	Cytokine signalling in immune system	57.97	29 (760)	FLT3, FOS, IL1B, OAS1, LCK, CSK, IL6, ITGAM, UBA52, IL2RB, GBP1, SÝK, MT2A, 112, IL7R, SAMHD1, HLADQB1, HLAF, CDKN1A, GBP5, RPS27A, SOCS3, PLCGI, EGF, IRF2, IL3RA, CD4, FYN, MCL1

#### 3.4.4 Phenotypic and genetic relationships with the disease

The phenotypic abnormalities provided by Human phenotype ontology (HPO) of DEGs from AML showed the phenotypic decrease including T cell count, abnormality of the immune system and cellular immune system morphology, abnormal lymphocyte and leukocyte morphology and leukopenia (decreased WBC count) etc. Based on the number of overlapping genes and the clinical significance of the gene-disease relation, disease matching scores were obtained. The top gene-disease relationship showed the association of many genes with leukemia, especially acute myeloid and acute lymphoblastic. This also shows the importance of structural variation and hence further recalculation based on filtering found that mutation in the five genes FLT3, PAX5, ETV6, RUNX1 and MPO have their correlation with structural variation in AML.

### 3.5 Variant analysis on immune checkpoint molecules of AML

**Figure 4:**
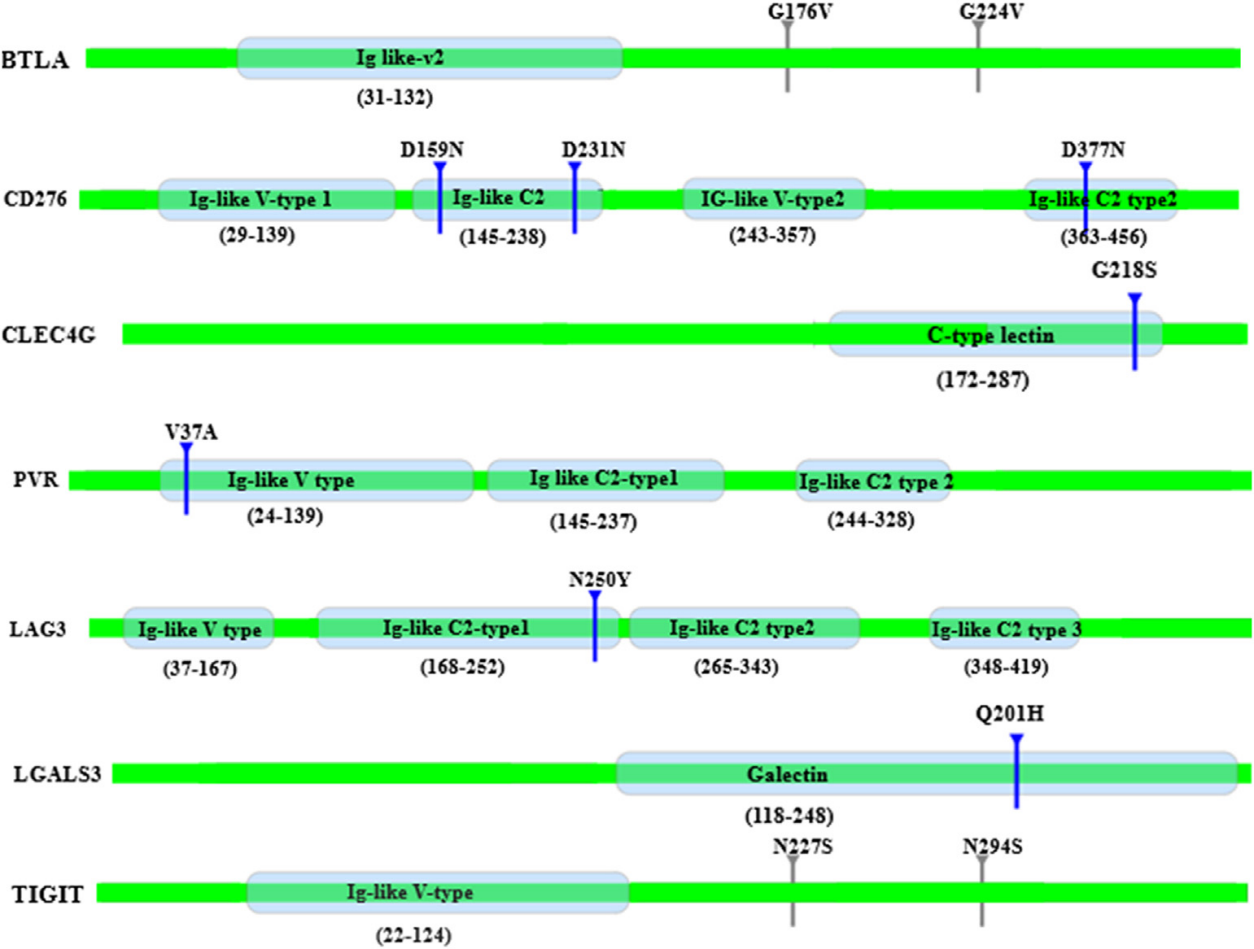
Deleterious mutations observed in immune checkpoint molecules. Grey color regions (in TIGIT and BTLA) display mutation other than domain regions. CD276, CLEC4G, PVR, LAG3 and LGALS3 show substitutions in their domain region.

The screened genes from DEGs and PPI network analysis were also further subjected for analysis of variants. Detailed investigation on the variants of immune checkpoint molecules were carried out due to their importance by this study identified the amino acid substitutions at the important domains which is shown in Figure 4, predicted as disease-causing ability. This found the non-synonymous variants in co-stimulatory checkpoint targets CD28, CD226, PVR (CD155) and the co-inhibitory targets BTLA, LAG3, B7-H3 (CD276), CEACAM1, HAVCR2, LGALS9, PD1, PDL1 (CD274) and TIGIT.

### 3.6 Validation by survival analysis

**Figure 5:**
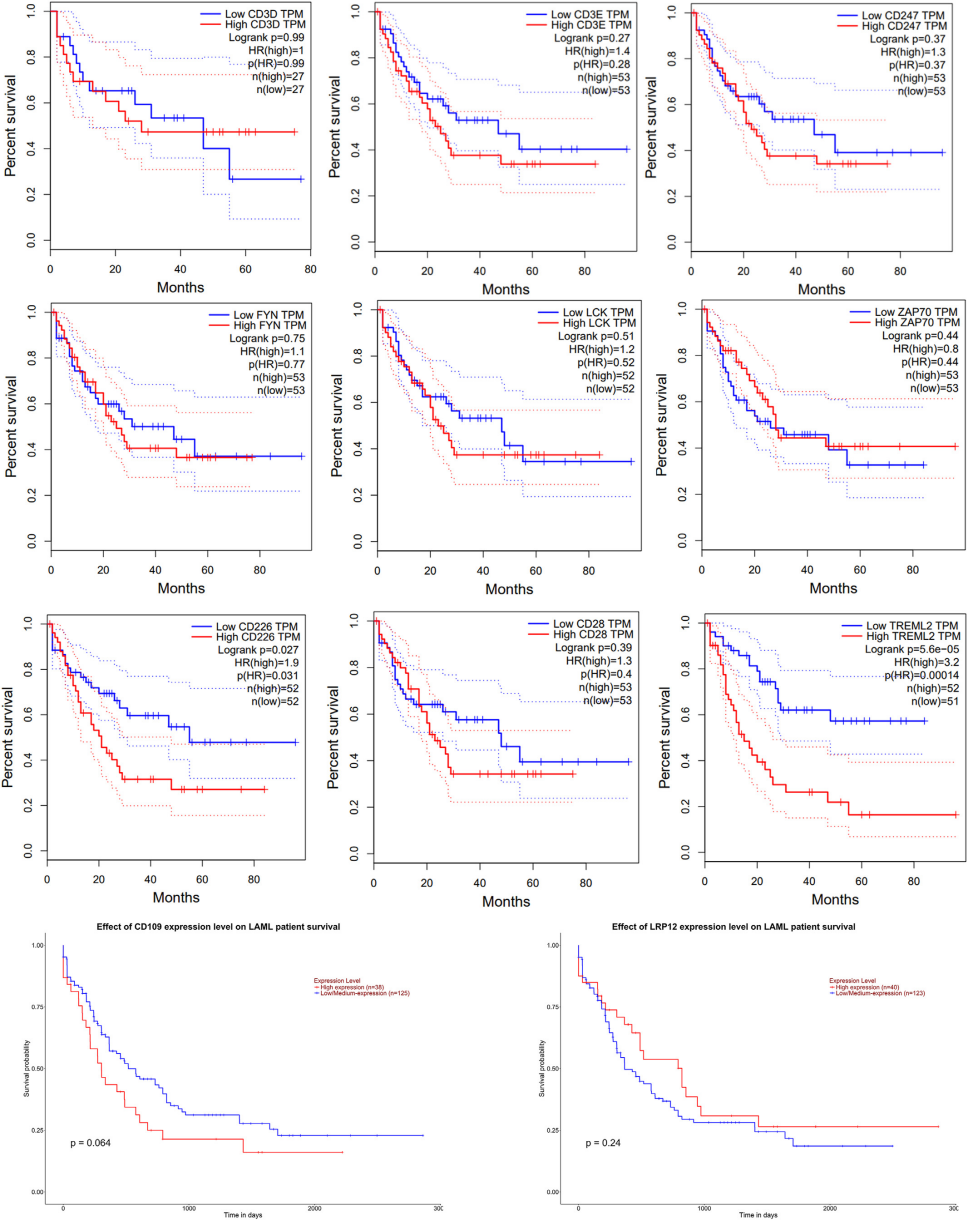
Kaplan-Meier survival curves of the DEGs related to TCR signaling. CD3D, CD3E, CD247, FYN, LCK, ZAP70, CD226, CD28. TREML2, CD109 and LRP12 are shown. TPM-transcripts per million. HR- Hazards score, which is calculated based on Cox model.

Survival analysis identified some genes related to immune response in particular TCR signalling to be correlated with AML patient survival. Log-rank overall survival curves showed that the gene expression pattern of CD3D, CD3E, CD247, FYN, LCK, ZAP70, CD226, CD28 and TREML2 were significantly associated with overall survival as depicted in Figure 5.

### 3.7 CD109 and LRP12 as possible biomarker prediction and validation

The cell surface protein products of the deregulated genes of AML may withstand the external cell signaling mechanisms in AML and thus should be further studied to determine their diagnostic and prognostic value in the patients. The sequences of the significant cell surface proteins extracted from the previous step of PPI networks were subjected to secretome analysis by SignalP [26]. By analysing the DEGs localized in the membrane, seven biomarkers including CD109, LRP12, EGFL7, FURIN, GAS6, LDLR and MMRN1 were predicted to have the ability to act as biomarker (Table 5). Based on databases and available literature search, CD109 and LRP12 were predicted as possible biomarkers for AML which is the first report from this study [27]. CD109 and LRP12 was validated using KM survival analysis and were significantly associated with overall survival as shown in Figure 5.

**Table 5:** Identified candidate biomarkers of Acute Myeloid Leukemia.

Sl. No	Gene	Description	Subcellular localization
1	CD109	Cluster of Differentiation 109	Plasma membrane
2	LRP12	LDLR-related protein 12	Plasma membrane
3	FURIN	Furin, Membrane Associated Receptor Protein	Extracellular
4	GAS6	Growth arrest specific 6	Extracellular
5	LDLR	Low-density lipoprotein receptor	External side of plasma membrane
6	EGFL7	Epithelial growth factor-like 7	Extracellular
7	MMRN1	Multimerin 1	Extracellular

### 3.8 CD109 and LRP 12 expressions were demonstrated by western blot analysis

**Figure 6:**
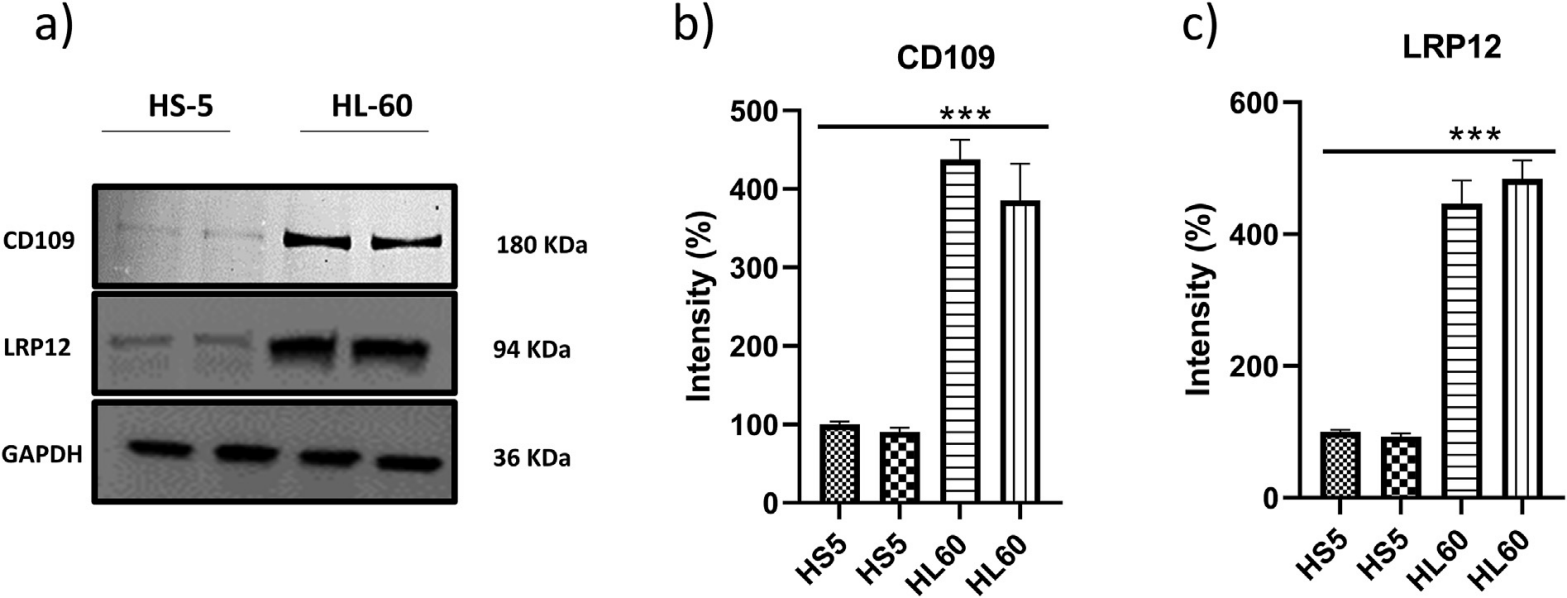
CD109 and LRP12 are overexpressed/upregulated in HL60 acute myeloid leukaemia (AML) cell line compared to human bone marrow-derived stromal cell line HS-5, in vitro. a) Representative western blots of CD109 and LRP12 expression; b and c) shows the protein expression of CD109 and LRP12 in HS-5 and HL-60 cells from the western blot quantifications, normalized to GAPDH. ∗∗∗p < 0.001 compared with the control group.

**Fig_S2:**
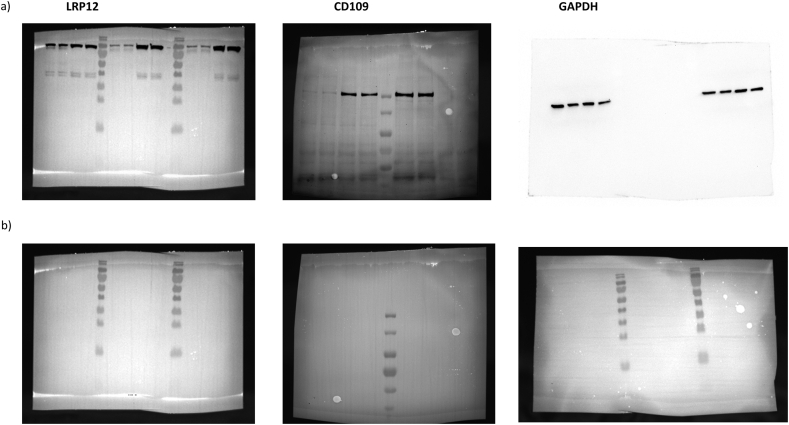


The western blot results showed CD109 and LRP12 proteins expression were upregulated in HL60 cells from normal cell HS5 (Figure 6). Images of the uncropped original western blot are provided in Supplementary Figure S2.

## 4 Discussion

Despite the fact that, alteration in immune system and immune-related components have been identified as one of the hallmarks of cancer and the effect of immune microenvironment in survival and response to treatment in solid tumours, their impact on leukemia is not known fully [28, 29]. Here, we compared the gene expression profiles of AML and normal samples to assess their differential expression in cases of haematological malignancies particularly, AML. AML is considered a heterogenous genomic landscape caused due to numerous genetic modifications, making disease classification and management complicated [2]. Even though studies reported significant genes in AML, their relation to pathogenesis and prognosis has not been completely elucidated. Hence an integrative analysis based on multi-genomic data is essential to determine the relationship between specific genes and cancer progression. By mining the high throughput RNA-Seq data the significantly differentially expressed genes were identified.

In order to investigate the molecular mechanism behind the pathogenesis of AML, our data revealed a total of 655 DEGs with up-regulated and down-regulated genes. EGFL7, MCL1, IGHG1, CD109 and GNA15 were the top five upregulated genes and CD3E, GNLY, IL32, FGFBP2 and FCMR were found to be the top five downregulated genes. This highlights CD109, a glycosylphosphatidylinositol (GPI)-anchored protein, which was recently reported its association with different tumor entities and a possible future diagnostic marker linked to reduced patient survival. Also, different cell signalling pathways including the TGFβ, JAK-STAT3, YAP/TAZ, and EGFR/AKT/mTOR were proposed as targets for CD109 interference pathways [30, 31, 32]. The present study reveals the CD109 as a possible biomarker for AML. The interactive relationships among all DEGs by protein-protein interaction (PPI) network found out sixteen genes with up or down regulation pattern with TP53, PTPRC and AKT1 as top three hub genes. Here, TP53 mutation is detected in up to 75% of patients, while patients who harbor co-occurring mutations show a lower incidence of mutations in several AML-related genes such as NPM1, FLT3, IDH1, IDH2, WT1, DNMT3A, RUNX1, and RAS [33, 34]. Protein tyrosine phosphatase receptor type C (PTPRC), a subgroup of Protein tyrosine phosphatases, which plays a major role in regulation of cell-signalling and controls cell growth, differentiation, apoptosis, survival, migration and invasion [35] was majorly altered in the AML which is an essential regulator of T and B cell antigen receptor-mediated activation [36]. A recent report by Saint-Paul et al found that PTPRC also known as CD45 is involved in the progression of AML through modification in plasma membrane bound lipid rafts, cholesterol and glycosphingo lipid enriched patches which is correlated from our results [37].

To gain a better understanding of the underlying biological functions and pathways associated with AML gene signature, we utilized ClueGO [38], which is a functional gene ontology analysis tool that integrates several gene-set enrichment databases, including KEGG, REACTOME pathway database annotations, and the GO consortium database, to create a comprehensive GO/pathway term network. Further PPI network with MCODE screened out 106 DEGs including genes related to immune response and T-cell receptor (TCR) signalling for AML. Likewise, Han et al. (2020) reported top three hub genes of PPI network of FANCI, POSTN, IFIH1, ZMYND10, PACRG and POU2AF1 for nasopharyngeal carcinoma biomarkers using STRING database PPI network construction with MCODE for module analysis [39]. In the present study, the number of proteins associated with immune response, was high, which was consistent with the GO analysis and DEGs identification analysis, suggesting immune response may play an important role in the pathogenesis of AML.

Further, GeneAnalytics predicted twenty super pathways and the innate immune system pathway presented the highest match score top pathway. Current researches in cancer mainly focus on the identification of mechanisms that inhibit the binding of T cells with its ligands, stimulating tolerance induction, which permits the positioning of T cells to fight against the cancer cells. The activation of T cell involves several extracellular stimulatory molecules mediated primarily by TCR complex and precise T cell regulation is essential for maintaining immune homeostasis [40]. TCR complex containing TCR α/β chains, a CD3E chain and a CD3 co-receptor, associated through hydrophobic interactions has a tightly controlled assembly and expression within cells [41]. CD3E is an essential part of the TCR signalling pathway and its downregulation has been reported in several conditions associated with inflammation [42]. Studies reported the association of CD3E levels with T-cell response and proliferation [42, 43]. Low CD3E leads to reduced immune responses including a decrease in cell proliferation and cytokine production [44].

Mutation in TRAC (T cell receptor α constant) gene impairs surface expression of TCR αβ complex [45]. As most of the genes downstream of TRAC were found to be down-regulated, we believe that it might be due to the lack of surface expression of TCR αβ due to mutation in TRAC gene. Hence, we searched for its mutation specific to AML from the variant data, which found a synonymous mutation at position 81 (i.e., I81I) of the amino acid sequence. This queries whether this mutation affects downstream phosphorylation events as a single synonymous mutation prevents phosphorylation and decides stability of TP53, a tumor suppressor gene [46].

Downregulation of genes involving TCR signalling, also insists to search for the factors which impair the interaction of the antigen-presenting cells with the T-cell and affects T-cell activation. A decrease in expression of MHC molecules suggests a lack of antigen presentation to the T cells. Naive T-cell activation involves the stimulation of TCR by an MHC-peptide complex and co-stimulatory signalling by co-stimulatory receptors with their respective ligands on antigen-presenting cells (APCs) [47]. T-cell co-signalling receptors (immune checkpoints) either positively (co-stimulatory) or negatively (co-inhibitory) regulate TCR driven signals, thus activating T-cell. As these receptors play a vital role in T-cell biology, the expression of these co-receptors and their ligands are firmly controlled in T-cells and the tissue micro-environment [48]. Effective T-cell activation needs both TCR stimulation and co-stimulation by checkpoint proteins. The co-stimulatory proteins CD28 and CD226 were found to be downregulated in the present findings analysed by DEGs, PPI network and variant analysis. CD28, the major costimulatory receptor for T-cell activation affects the expression of some genes varied by TCR stimulation alone. CD28 signalling enhances the expression of CD226 [49], suggesting lack of CD28 expression might have reduced the expression of CD226. The decrease in CD28 might be due to the competence of an inhibitory checkpoint molecule CTLA-4, which shares the common B7-related ligands CD80 and CD86. Also, the affinity of CTLA-4 with B7 is greater than that of CD28 with B7, which allows CTLA-4 to outcompete and inhibit co-stimulatory CD28/B7 interactions [50]. Another study reported the decreased expression of immune checkpoint receptors in AML compared to other types of leukaemia which coincides with our results too [51]. However, they noticed varied immunologic phenomena in different AML patients. Overall, this study identified the decreased expression of immune response-related genes, suggesting the incapability of the normal functioning of immune system in AML.

Survival analysis revealed the association of deregulated genes related to TCR signalling with poor overall survival. ZAP70 has been reported to have a significant association with poor overall survival in chronic lymphocytic leukaemia [52, 53]. Okamoto et al. [52], and Robinson et al. [54], suggested the involvement of Src family kinases especially LYN and LCK in FLT3-induced cell survival. Our analysis also shows the involvement of Src kinases LCK and FYN in poor clinical outcomes of AML patients. Low CD3D expression was correlated with increasing clinical stage in colon adenocarcinoma and its increased expression showed better clinical outcome [55]. Lower CD226 and high TIGIT may predict poor prognosis in AML patients and the imbalance in TIGIT/CD226 axis may be the immune checkpoint barrier responsible for T-cell immune dysfunction [56]. Li et al. [57], reported the reduced survival time in correlation with high TREML2 (aka. TLT2). In contrast, our study showed a slight decrease in expression of TREML2, which too correlated with a poor survival rate.

**Figure 7:**
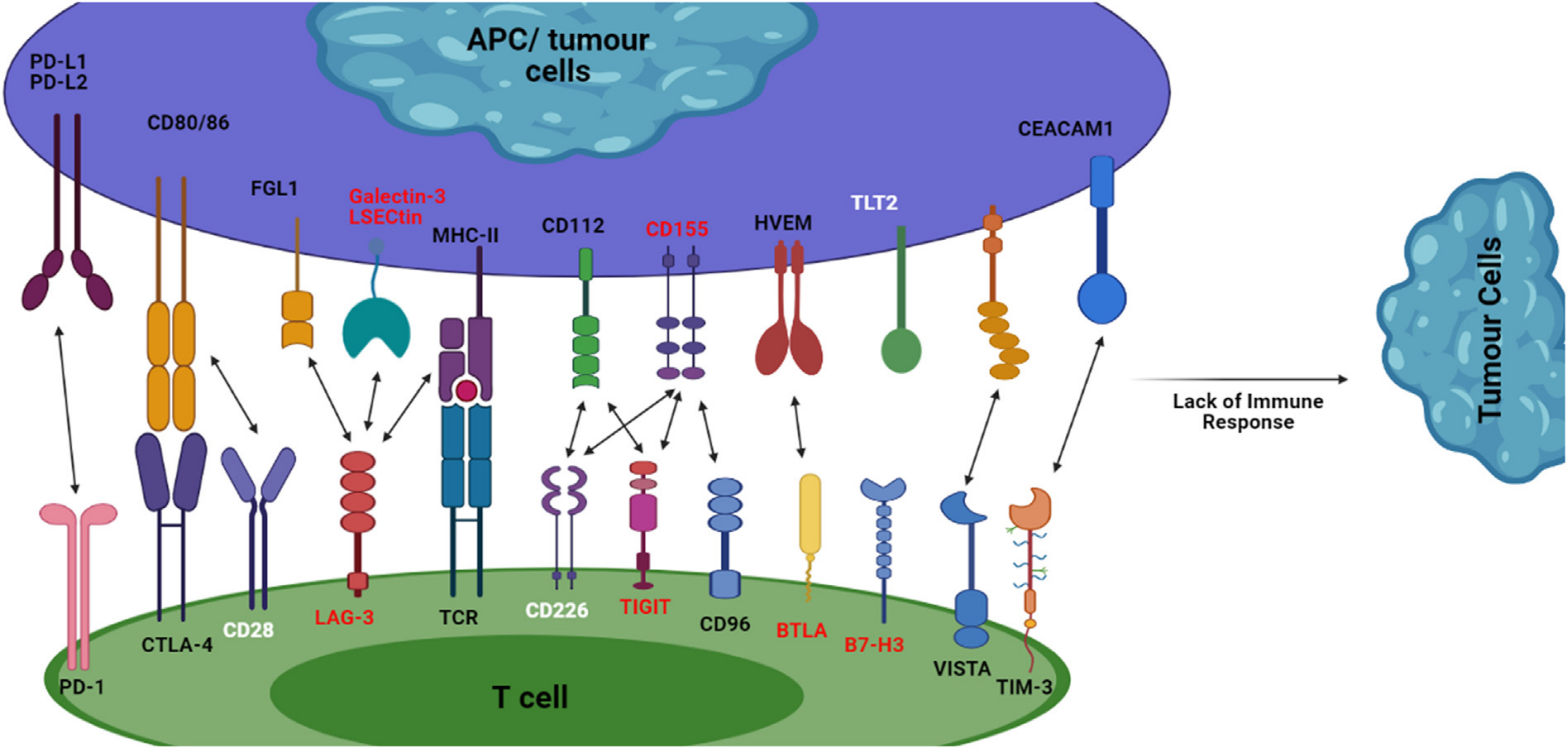
Proposed model of Co-stimulatory and Co-inhibitory immune checkpoint molecules in AML. The mechanism is activated by the presentation of antigen (Red circle) by MHC-II of the APC/tumour cells to the TCR of T cell. Other co-regulatory interactions occur simultaneously as depicted. The down regulated genes are labelled white (CD28, CD226 and TLT2) and red label denotes those which show deleterious mutation in the domain region. CTLA-4 competes with CD28, thereby sending negative signals. Mutation in the domain regions of B7-H3 might disrupt its interaction with TLT2. Hence all the positive signals might be prohibited and activation of negative signals resulting in inhibition of immune response.

As the immune response is found to be downregulated in our study, aiming to gain further insights regarding the lack of T-cell activation, we searched for the exonic variants specific to AML associated with the immune checkpoint molecules responsible for T-cell activation. This found the non-synonymous variants in co-stimulatory checkpoint targets CD28, CD226, PVR (CD155) and the co-inhibitory targets BTLA, LAG3, B7-H3 (CD276), CEACAM1, HAVCR2, LGALS9, PD1, PDL1 (CD274), TIGIT. Based on the results we proposed a mechanism of T cell activation in AML (Figure 7), discussed below as depicted. Analysing possible impact of the identified amino acid changes on their structure and function predicted the variants associated with BTLA, B7-H3, CLEC4G, LAG3, LGALS3, PVR and TIGIT to be disease-causing. Further investigation revealed the amino acid substitutions in domain regions of B7-H3, CLEC4G, LAG3, LGALS3 and PVR. Only a synonymous variant V65V (coded by C195T) was observed in CD28. B7-H3, a type I transmembrane protein is believed to express on immune cells playing a co-stimulatory role, but their function on immune cells is unclear [58, 59]. More than 20 variants were observed for the gene and three amino acid substitutions namely D159N (n.G475A), D231N (n.G691A) and D377N (n.G1129A) in various domains were predicted deleterious. Hashiguchi et al. [60, 61] reported TLT2 (Trem-like transcript 2; TREML2) as the binding partner of B7-H3, however, no others studies have not yet confirmed it. Downregulation of TLT2 might be due to the inability of the ligand to recognize the receptor B7-H3, because of the substitutions in its Ig-like type 1 and 2 domains. Comparatively, numerous variations have been observed for B7-H3 in our study, which might disturb its folding pattern and function thereby limiting our knowledge in finding its perfect ligand. Lymphocyte activation gene 3 (LAG3; CD223) is a co-inhibitory molecule expressed on activated T-cells, Tregs, NK cells, B cells and dendritic cells [62]. Structurally, LAG3 resembles a CD4 co-receptor nevertheless, interacts it with MHC-II with increased affinity than CD4. LSECtin, an alternative ligand of LAG3 regulates LAG3 expressing CD8 T cells and NK cells. Moreover, enhanced expression of LAG3 on CD8 T cells reveals their dysfunction in anti-tumour activity, thus being an important target for blocking checkpoint in cancer immunotherapy [63]. A non-synonymous mutation (N250Y) in exon 4 of LAG3 residing in the region of its interaction with FGL1 was observed. Change in residue occupying the immunoglobulin-like domain, observed in B7-H3, LAG3 and PVR which might disturb their interaction with respective binding molecules, an Ig-like domain usually requires its intact domain for binding with another molecule. We observed a non-synonymous mutation namely A562G corresponding to T188A at the Ig-2 domain of CD226. As this domain generally interacts with the ligand of CD226 during T-cell activation, the introduction of the hydrophobic residue might disturb this interaction.

Currently, most of the blood clinical tests are based on secreted proteins, which can be used as diagnostic or prognostic markers. By analysing the DEGs localized in the extracellular region, CD109, LRP12, EGFL7, FURIN, GAS6, LDLR and MMRN1 were predicted to have the ability to act as biomarker because of their secretory role. Studies reported the identified biomarkers to have their role in AML survival. EGFL7, a secreted angiogenic factor as a biomarker coincides with study by Cheng et al. [64], highlighting the poor prognosis with increased EGFL7 expression in AML. FURIN, a potential oncogene can target several oncogenic pathways simultaneously, which would be beneficial in improving the efficiency of cancer treatments. Increased expression of Gas6 correlates with shorter overall survival in AML patients [65, 66]. MMRN1 had also been suggested as a predictive biomarker in AML. LDLRs (Low-density lipoprotein receptors) showed altered expression in several cancers including leukemia [64, 67]. Floeth et al. [68] anticipated the contribution of LDLR in chemotherapy resistance and suggested it to be an independent adverse prognostic factor in AML. Orentas et al. [69] reported the increased expression of LRP12, another Low-density lipoprotein in pediatric lymphoid leukemia [70]. However, no studies reported their overexpression in AML to our knowledge. Our *in vitro* analytical validation was carried out in AML cell line and HL 60 cells and human bone marrow-derived stromal cell line HS 5 which are routinely used for the study of AML [71, 72]. HL-60 was studied and characterized previously for Acute myeloid leukemia studies and the expression of pattern of CD109 and LRP12 could confirm its validation. The western blots of CD109 and LRP12 expression confirms the protein expression of CD109 and LRP12 in HL-60 is higher than HS-5 and cells which was normalized to GAPDH. CD109, a negative regulator of TGF-β signalling is a possible prognostic biomarker in epithelioid sarcoma [73] and penile squamous cell carcinoma [74]. However, no studies reported the biomarker potential of CD109, LRP12 in AML. Thus, revealing novel biomarkers may contribute to better understanding the molecular basis of AML, which may play an essential role in the diagnosis of AML, leukaemia residual monitoring, prognostic stratification, as well as the possibility of targeted drug development. However, the limitation of the present study is the small sample size of RNA-seq. To ensure greater reliability of the present observations and assumptions, the sample should be expanded for further research in the future. Clinical samples and experimental validation should be utilized to verify the prognostic predictive role of CD109 and LRP12 mRNA and protein in AML.

## 5 Conclusion

In conclusion, this study addressed the genes and pathways involved in the transcriptomic deregulations due to AML. Various bioinformatic tools have been utilized to mining the transcriptomes for DEGs, PPI networks, gene ontology, KEGG pathway, variant analysis and secretome analyses to unmask the heterogenic nature of AML. Deregulation of genes related to immune response in particular TCR signalling pathway is found to be emphasized based on pathway, network and functional analyses. TP53, PTPRC and AKT1 were identified as top three hub genes for AML. CD109, LRP12, EGFL7, FURIN, GAS6, LDLR, MMRN1 and PTK7 were predicted to have the ability to act as possible biomarkers based on their secretory function from our study. Moreover, this study emphasizes the significance of the genes CD3D, CD3E, CD247, FYN, LCK, ZAP70, CD226, CD28 and TREML2 were associated with overall survival. The study revealed the mechanism of decrease in immune response due to the downregulation of co-stimulatory immune molecules, whereas no alteration was observed in co-inhibitory molecules thus affecting the maintenance of proper immune homeostasis in AML. Further studies on these mutations and their impact on interaction with respective partners and a detailed understanding of these mechanisms in combination with advanced therapeutic approaches will be beneficial in designing potential clinical applications for AML.

## Declarations

### Author contribution statement

EbyNesar StellaGlory Deepak Shyl: Conceived and designed the experiments; Performed the experiments; Analyzed and interpreted the data; Wrote the paper.

Beutline Malgija; Appadurai Muthamil Iniyan: Conceived and designed the experiments; Analyzed and interpreted the data; Wrote the paper.

Ramasamy Mahendran: Analyzed and interpreted the data.

Samuel Gnana Prakash Vincent: Conceived and designed the experiments; Contributed reagents, materials, analysis tools or data.

### Funding statement

This work was supported by the 10.13039/501100006143Department of Science and Technology - Fund for Improvement of S&T Infrastructure (DST-FIST), Govt. of India [F.No. SR/FST/LSI-559/2013].

### Data availability statement

Data will be made available on request.

### Declaration of interests statement

The authors declare no conflict of interest.

### Additional information

No additional information is available for this paper.

## Figures and Tables

**Figure:**
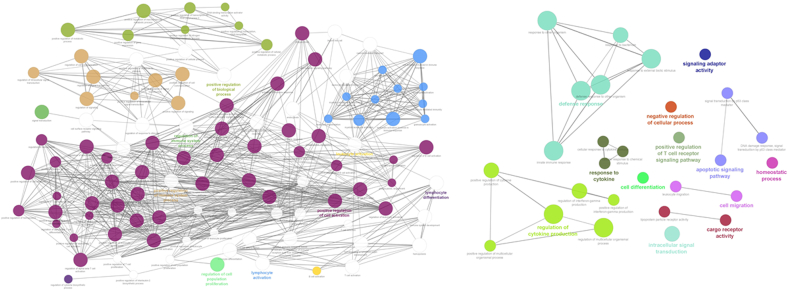
Fig_S1

## Appendix A Supplementary data

Suppl Table Fig Suppl Table Fig
