# Supplementary material for: Mining of transcriptome identifies CD109 and LRP12 as possible biomarkers and deregulation mechanism of T cell receptor pathway in Acute Myeloid Leukemia
Source: Heliyon. 2022 Oct 18;8(10):e11123. doi: 10.1016/j.heliyon.2022.e11123 (PMC9589179; doi:10.1016/j.heliyon.2022.e11123)
Supplement: Suppl Table Fig [file mmc1.docx]

**Supplementary Information**

Mining of transcriptome identifies CD109 and LRP12 as possible biomarkers and deregulation mechanism of T cell receptor pathway in Acute Myeloid Leukemia

**Table S1. List of RNA-seq samples used for the analysis.**

| **Sample** | **Condition** | **Tissue** | **Gender** | **Age** |
| --- | --- | --- | --- | --- |
| ERR2019340 | Acute myeloid leukemia | bone marrow | Male | 65 |
| ERR2019341 | Acute myeloid leukemia | blood cell | Male | 46 |
| ERR2019339 | Acute myeloid leukemia | bone marrow | Female | 53 |
| SRR10257577 | Acute myeloid leukemia | Bone marrow | Female | 51 |
| SRR10257576 | Acute myeloid leukemia | Bone marrow | Male | 56 |
| SRR6433220 | Acute myeloid leukemia | Bone marrow | Male | 75 |
| SRR6433221 | Acute myeloid leukemia | Bone marrow | Male | 66 |
| SRR6433222 | Acute myeloid leukemia | Bone marrow | Female | 30 |
| SRR10257584 | Normal | Bone marrow | Male | 48 |
| SRR10257582 | Normal | Bone marrow | Female | 50 |
| SRR10257583 | Normal | Bone marrow | Female | 50 |
| SRR6433218 | Normal | Peripheral blood | Male | 26 |
| SRR6433219 | Normal | Peripheral blood | Male | 30 |
| SRR5682965 | Normal | Peripheral blood | Male | 26 |
| SRR5682966 | Normal | Peripheral blood | Male | 26 |
| SRR10257585 | Normal | Bone marrow | Male | 34 |

**Table S2.** **Gene Ontology terms biological process and molecular functions related to AML.**

| **S.No** | **Name** | **Score** | **Matched genes (Total genes)** |
| --- | --- | --- | --- |
| ***Molecular function*** | | | |
| 1. | Protein binding | 48.61 | 93 (11207) |
| 2. | Non-membrane spanning protein tyrosine kinase activity | 33.22 | 8 (46) |
| 3. | Identical protein binding | 29.19 | 26 (1371) |
| 4. | Protein tyrosine kinase activity | 26.05 | 9 (126) |
| 5. | Phosphotyrosine residue binding | 23.68 | 6 (42) |
| ***Biological process*** | | | |
| 1. | T cell receptor signalling pathway | 41.44 | 14 (179) |
| 2. | T cell differentiation | 40.45 | 9 (40) |
| 3. | T cell activation | 37.86 | 9 (49) |
| 4. | Immune system process | 37.77 | 23 (788) |
| 5. | Membrane organization | 37.45 | 12 (138) |
| 6. | Cytokine-mediated signalling pathway | 35.45 | 15 (297) |
| 7. | Neutrophil degranulation | 35.15 | 18 (484) |
| 8. | Transmembrane receptor protein tyrosine kinase signalling pathway | 34.15 | 11 (129) |
| 8. | Cellular protein metabolic process | 33.14 | 13 (226) |
| 9. | Post-translational protein modification | 32.43 | 15 (346) |
| 10. | Cell surface receptor signalling pathway | 30.68 | 14 (317) |
| 11. | Peptidyl-tyrosine autophosphorylation | 30.38 | 7 (36) |
| 12. | Positive regulation of Alpha-beta T cell proliferation | 29.66 | 5 (9) |
| 13. | Positive regulation of T cell proliferation | 29.52 | 8 (64) |


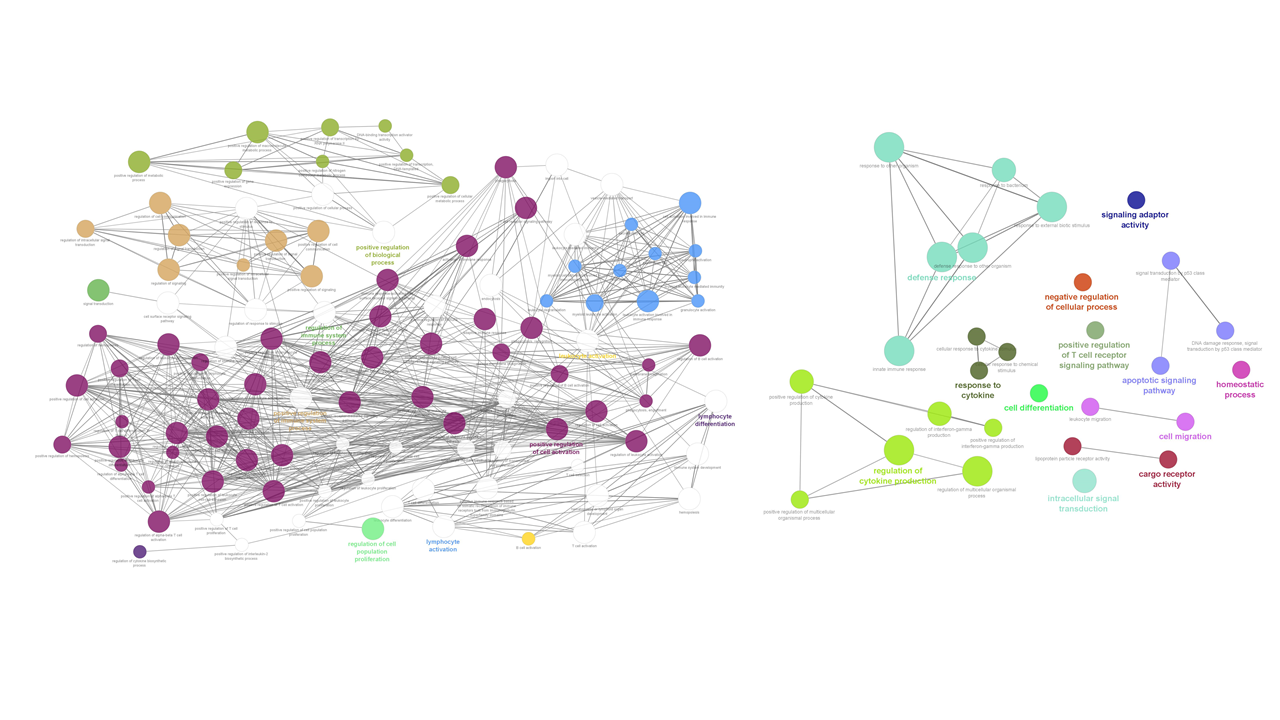


**Figure S1. Simplified networks of related statistically significant enriched GO terms using the Cytoscape add-on ClueGO**. The network was made from the DEGs of AML. ClueGO allows enrichment analysis and the collapsing of GO terms into parent categories for each comparison. Each of the terms is statistically significant (Benjamini-Hochberg correction <0.05). The filled colored circles (nodes) represent each statistically significant enriched parent GO term. The lines (edges) between the nodes show that there are overlapping genes within terms. The different sizes of the nodes relate to the degree of enrichment of the nodes. The network was automatically visualized using the organic layout algorithm. Only the label of the most significant term is highlighted.


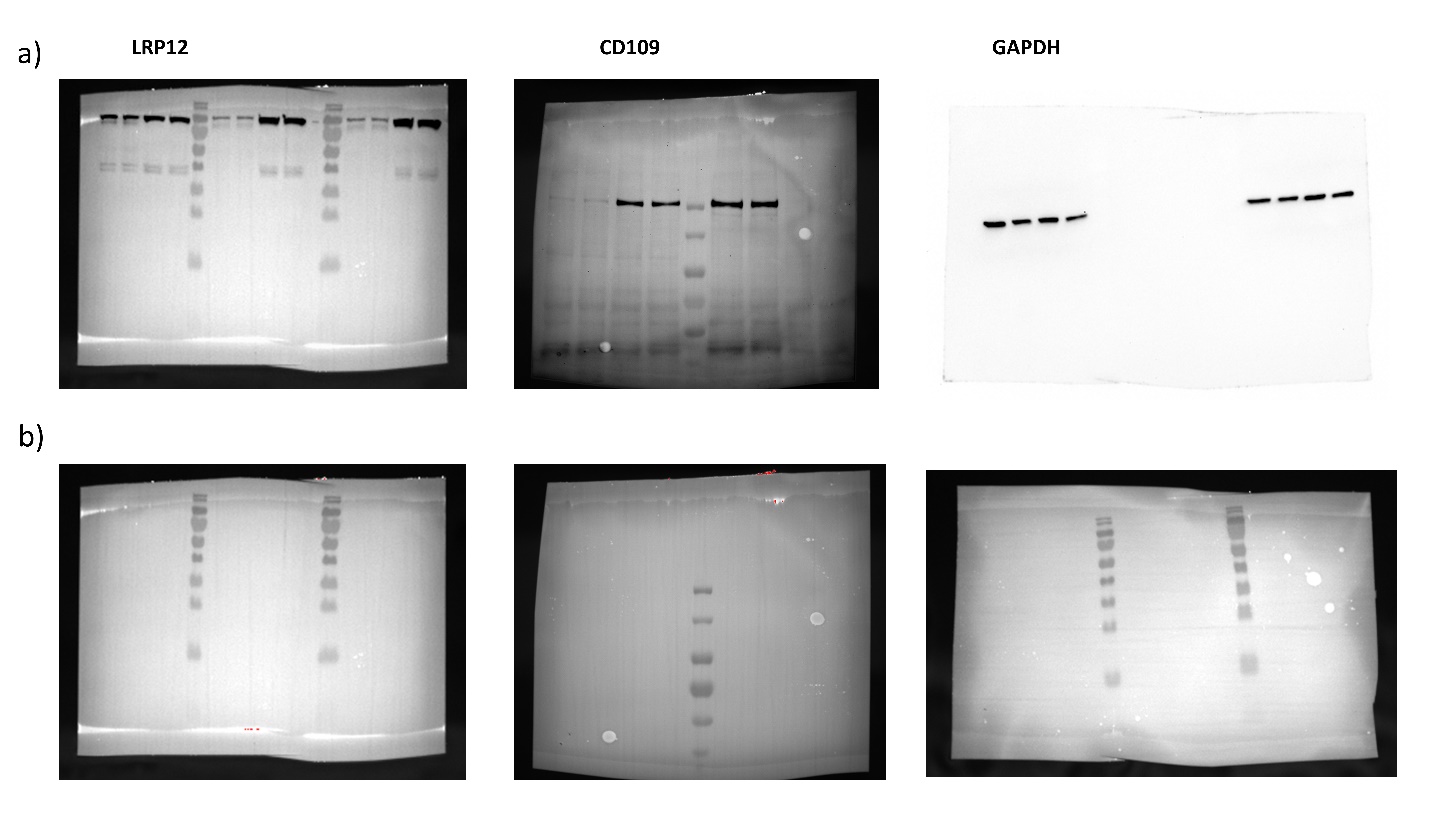


**Figure S2. Original western blot images from figure 6.** (a) Uncropped western blot images containing molecular weight markers LRP12, CD109 and GAPDH are shown. (b) Staining of membranes after protein transfer.
